# Clinical Medicine

**Published:** 1894-12

**Authors:** 


### Reviews of Books.

*Clinical Medicine.* By JUDSON S. BURY, M.D. Pp. xx., 468. London: CHARLES GRIFFIN & COMPANY, LIMITED. 1894.

We cannot attempt in a short review to give even an outline of the large amount of information that is contained in this work. There is now an almost embarrassing number of books on clinical diagnosis to choose from; but we think that this is one of the best, and we have no hesitation in cordially recommending it. It is well and clearly written; its style is interesting, and it is calculated to encourage the clinical student to undertake more exact and thorough investigation of his cases. The illustrations are abundant, excellent, and judiciously selected: those illustrating the deformities of the hands and feet in peripheral neuritis taken from the late Dr. Ross's book are particularly good; there are plenty of well-executed diagrams, and a number of sphygmograms. We think a short account of two or three of the best cardiographs in use might have been given.

The book essentially consists of a detailed account of the methods of examination of all the organs of the body, of the excretions, and of the variations from the normal in both organs and excretions that are met with in disease. The descriptions of the methods of physical diagnosis, of chemical and bacteriological examination of the fluids and excretions of the body, are everywhere clear, and the directions are so clear that they can be easily followed out. It is a most useful book both for the student in learning his work, and for the practitioner as a work of reference for help in all the multitudinous little points for investigation that occur in daily clinical practice. We will not single out any special section for praise, as all are good; but that on the nervous system, which is apt to be the weak point in books on clinical diagnosis, is particularly well done in this one.

The work is a perfect mine of information on clinical matters; it is very well arranged for ready-reference, and has that essential requirement for books of the kind, a full index. We congratulate the author that his labours, which must have been considerable, have had such a highly satisfactory result, and hope that his book will meet with the wide sale that it deserves to have. The printers and publishers have done their part of the work admirably.

